# Insights Into Enterovirus D68 Immunology: Unraveling the Mysteries of Host‐Pathogen Interactions

**DOI:** 10.1002/iid3.70117

**Published:** 2025-02-06

**Authors:** Asif Naeem, Muhammad Bashir Bello, Mohammad Bosaeed

**Affiliations:** ^1^ Infectious Diseases Research Department King Abdullah International Medical Research Center Riyadh Saudi Arabia

**Keywords:** EV‐d68, immunology, vaccine

## Abstract

**Background:**

Enterovirus D68 (EV‐D68) has emerged as a significant respiratory and neurological pathogen, particularly affecting children with severe respiratory illnesses and acute flaccid myelitis. Understanding the interaction between EV‐D68 and the host immune system is crucial for developing effective prevention and treatment strategies.

**Objectives:**

This review aims to examine the immune response to EV‐D68, its mechanisms of immune evasion, and the current progress in vaccine and antiviral development while identifying gaps in knowledge and future research directions.

**Methods:**

A comprehensive review of the literature was conducted, focusing on the innate and adaptive immune responses to EV‐D68, its strategies for immune evasion, and advancements in therapeutic interventions.

**Results:**

Pattern recognition receptors detect EV‐D68 and trigger antiviral defenses, including interferon production and activation of natural killer cells. B cells generate antibodies, while T cells coordinate a targeted response to the virus. EV‐D68 employs mechanisms such as antigenic variation and disruption of host antiviral pathways to evade immune detection. Progress in vaccine and antiviral research shows promise but remains in the early stages.

**Conclusions:**

EV‐D68 represents a complex and evolving public health challenge. Although the immune system mounts a robust response, the virus's ability to evade these defenses complicates efforts to control it. Continued research is essential to develop effective vaccines and antivirals and to address gaps in understanding its pathogenesis and immune interactions.

**Implications:**

A multidisciplinary approach is critical to improving diagnostic, preventive, and therapeutic strategies for EV‐D68, ensuring better preparedness for future outbreaks.

## Graphical abstract

This review provides a comprehensive overview of the immunological responses to Enterovirus D68, offering valuable insights for researchers, clinicians, and public health professionals working towards effective prevention and control strategies.

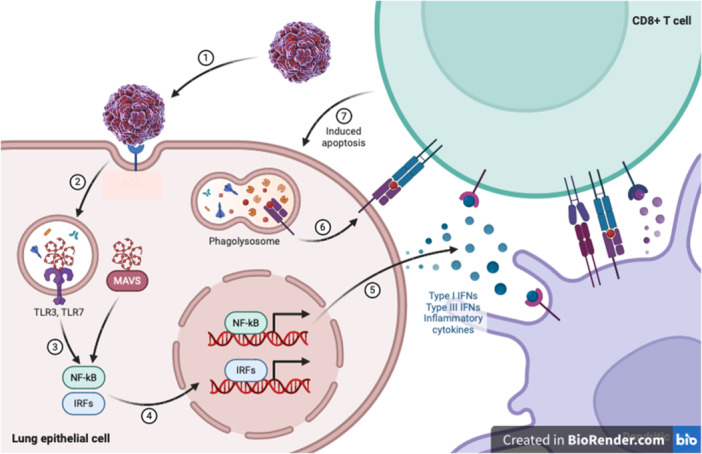

## 1 Introduction

Enteroviruses (EVs) are a group of viruses that fall under the Picornaviridae family. There are over 280 different viruses in this group that could infect humans. There are a total of 15 species in the genus, consisting of 12 Enterovirus species (A–L) and 3 Rhinovirus species (RV A–C) [1, 2]. Poliovirus serotypes 1–3 belong to the species C Enterovirus, while the other EVs are known as non‐polio enteroviruses (NPEVs). Out of the 15 NPEV species, 7 could infect humans. These NPEVs are widely recognized as common human pathogens with a global presence. Infections caused by NPEVs are especially prevalent in children. Some commonly circulating NPEVs include rhinoviruses, coxsackievirus A and B, EV‐D68, and EV‐A71 [1, 2].

Enterovirus D68 (EV‐D68) is a re‐emerging pathogen within the Picornaviridae family, known for causing respiratory illnesses and neurological conditions such as acute flaccid myelitis (AFM). First identified in California in 1962 [3], EV‐D68 was relatively rare until significant outbreaks in the United States in 2014, 2016, and 2018 highlighted its public health impact [4, 5, 6, 7, 8].

**Figure 1:**
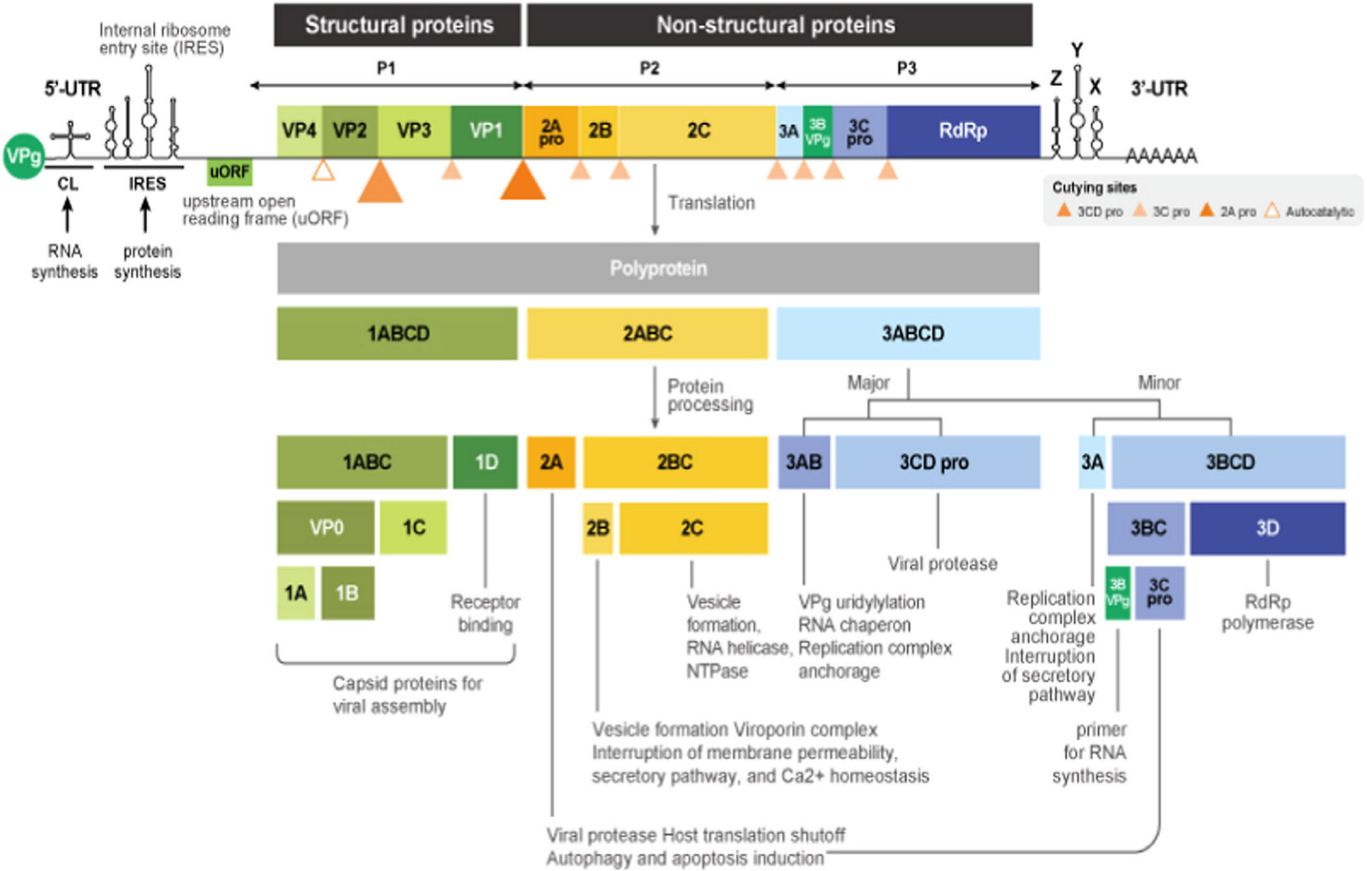
Genome organization of EV‐D68. The genome encodes for a single polyprotein that is post‐translationally processed into 11 mature proteins (Copyright GeneTex).

EV‐D68 is a non‐enveloped, positive‐sense single‐stranded RNA (ssRNA) virus. Its genome, approximately 7.5 kb in length, encodes a single polyprotein that is cleaved into structural and nonstructural proteins. The structural proteins (VP1, VP2, VP3, and VP4) form the viral capsid, crucial for protecting the viral RNA and mediating host cell entry. The nonstructural proteins (2A, 2B, 2C, 3A, 3B, 3C, and 3D) are involved in viral replication and modulation of the host's immune response (Figure 1) [9, 10].

**Figure 2:**
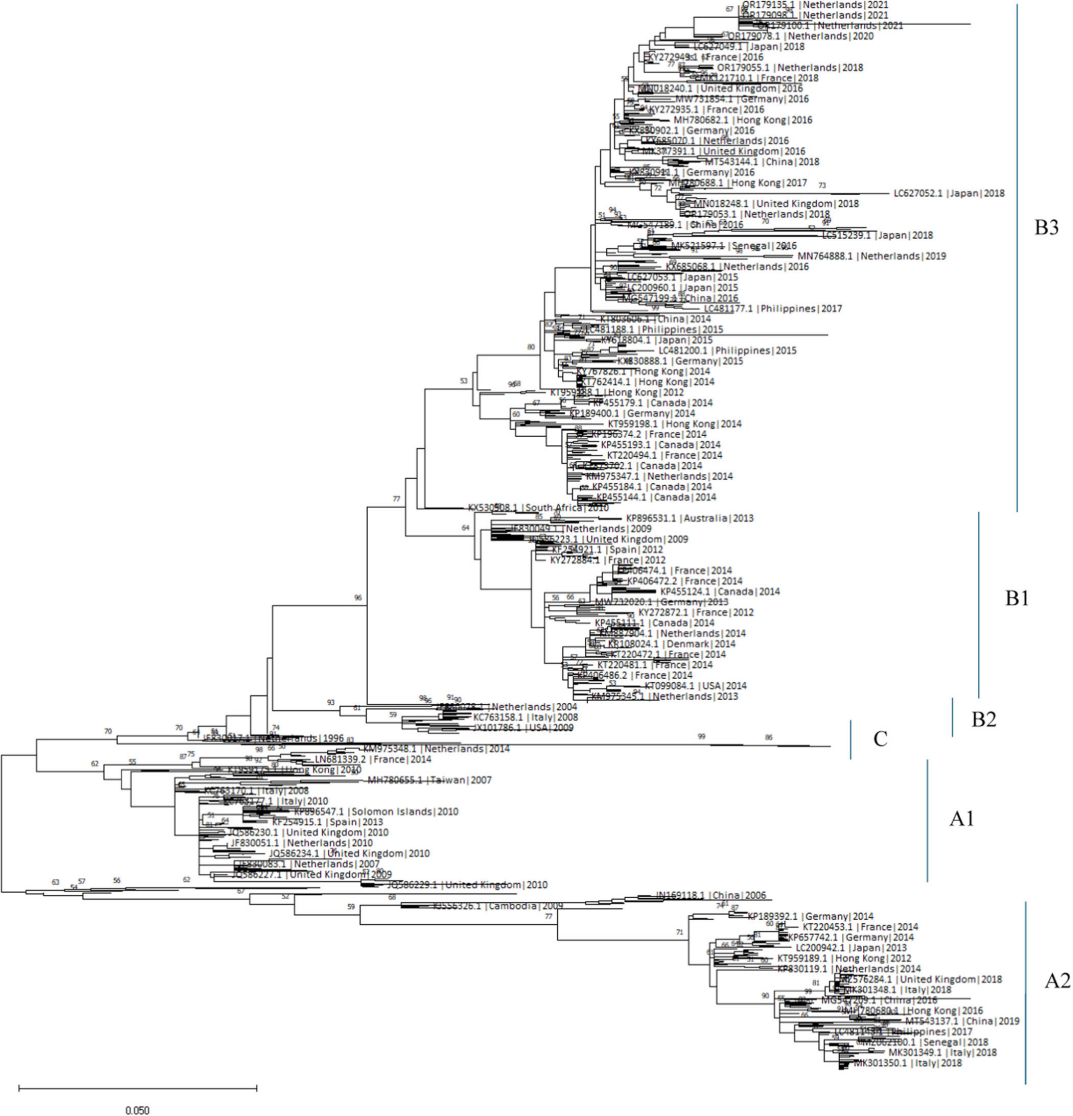
Evolutionary analysis by maximum likelihood method.

Historically associated with mild respiratory illnesses, EV‐D68 has garnered attention for its role in severe respiratory infections and AFM, particularly among children. The 2014 outbreak marked a turning point, with increased incidences of severe respiratory diseases and neurological complications [4, 5, 6, 8]. Subsequent outbreaks have shown a cyclical pattern, typically occurring in late summer and fall, suggesting a seasonal trend. EV‐D68 is phylogenetically divided into four subtypes: Clade A, Clade B, Clade C, and Clade D (Figure 2). The temporal and geographical distribution of EV‐D68 VP1 genetic sequence data shows that Clades A, B, and C are currently circulating in the United States, Europe, and Asia. Except for Clade C, most subtypes circulate together [11, 12, 13, 14, 15, 16]. A significant increase in EV‐D68 infections, with 139 cases identified across eight European countries between July 31 and October 14, 2021 has been reported. This surge aligns with the typical seasonality of EV‐D68 and is likely driven by the widespread reopening following COVID‐19 lockdowns [17]. Across 19 European countries, 58 institutes reported a total of 10,481 (6.8%) EV‐positive samples, of which 1004 (9.6%) were identified as EV‐D68, including 852 respiratory samples. Clinical data were available for 969 cases, with 78.9% of infections occurring in children aged 0–5 years and 37.9% of cases requiring hospitalization. Acute respiratory distress was the most common symptom (93.1%), followed by fever (49.4%). Neurological complications were observed in 6.4% of cases, including six diagnoses of AFM. Phylodynamic and phylogenetic analyses based on 694 sequences revealed the emergence of two novel B3‐derived lineages, without regional clustering. These data highlight a significant upsurge of EV‐D68 infections in Europe, marked by severe clinical outcomes and the emergence of new viral lineages [18].

Hixon et al. conducted a study to identify the cellular receptors used by EV‐D68 for entry into host cells. The researchers investigated whether sialic acid and the neuron‐specific receptor ICAM‐5, known receptors for other EVs, were utilized by EV‐D68. Their experiments involved using recombinant viruses and cell lines either deficient in sialic acid or lacking ICAM‐5 expression. By treating cells with neuraminidase to remove sialic acids, there was no significant reduction in EV‐D68 infection rates; they concluded that sialic acid is not a receptor for EV‐D68. Similarly, cells lacking ICAM‐5 expression did not show decreased susceptibility to EV‐D68 infection, indicating that ICAM‐5 is also not a receptor for the virus. Hixon et al.'s work suggests that EV‐D68 uses different receptors from those used by other well‐studied EVs, prompting further research to identify the actual receptors involved [19].

Rosenfeld et al. aimed to explore the receptor usage of EV‐D68 further, particularly focusing on its ability to infect neural cells, given the virus's association with AFM. EV‐D68 could infect a variety of cell types, including respiratory and neural cells, implying the involvement of widely expressed receptors or multiple receptors. Through similar experimental approaches to Hixon et al., Rosenfeld et al. confirmed that neither sialic acid nor ICAM‐5 is critical for EV‐D68 infection. They used gene knockout models and specific inhibitors to demonstrate that the absence of these molecules did not impede viral entry [20].

These studies collectively underscore the need to identify the precise receptors for EV‐D68, which remains a critical gap in understanding the virus's pathogenesis and developing targeted interventions.

Upon EV‐D68 infection, the host's innate immune system is the first line of defense [21]. Pattern recognition receptors (PRRs) such as Toll‐like receptors (TLRs) and RIG‐I‐like receptors (RLRs) detect viral RNA, triggering signaling pathways that lead to the production of Type I interferons (IFNs) and other pro‐inflammatory cytokines [22]. Type I interferons (IFN‐α and IFN‐β) play a critical role in establishing an antiviral state by inducing the expression of interferon‐stimulated genes (ISGs). These genes inhibit viral replication and spread, enhancing the antiviral defenses of neighboring cells [23]. The production of pro‐inflammatory cytokines such as IL‐6 and tumor necrosis factor (TNF)‐α helps recruit immune cells to the site of infection, promoting the clearance of the virus [24].

The adaptive immune response, involving B cells and T cells, is crucial for controlling and eventually clearing EV‐D68 infection. B cells produce neutralizing antibodies that bind to viral particles, preventing them from infecting host cells [25]. Serological studies have demonstrated the presence of EV‐D68‐specific antibodies in infected individuals [15, 26, 27]. CD8+ cytotoxic T cells recognize and destroy infected cells, while CD4+ helper T cells support the activity of B cells and cytotoxic T cells [27]. However, detailed characterization of T cell responses to EV‐D68 is still an area requiring further research.

This review aims to provide a comprehensive overview of the current knowledge of EV‐D68 immunology, highlighting key research areas and identifying gaps that need to be addressed. It, therefore, seeks to contribute to the ongoing efforts to develop targeted interventions and enhance public health outcomes related to EV‐D68 infections.

The evolutionary history was inferred by using the maximum likelihood method and general time reversible model. The tree with the highest log likelihood (−22876.39) is shown. The percentage of trees in which the associated taxa clustered together is shown above the branches. Initial tree(s) for the heuristic search were obtained automatically by applying Neighbor‐Join and BioNJ algorithms to a matrix of pairwise distances estimated using the maximum composite likelihood approach, and then selecting the topology with superior log likelihood value. A discrete Gamma distribution was used to model evolutionary rate differences among sites (five categories [+G, parameter = 0.4986]). The tree is drawn to scale, with branch lengths measured in the number of substitutions per site. This analysis involved 1220 nucleotide sequences. Evolutionary analyses were conducted in MEGA11.

### 1.1 Innate Immune Response

EV‐D68 interacts with the host's innate immune system in several ways. Although the understanding of these mechanisms is still evolving, current research highlights specific interactions and responses that play a crucial role in the host's defense against EV‐D68.

Upon infection, EV‐D68 is detected by the host's PRRs, such as TLRs and RLRs. These receptors recognize viral RNA and initiate signaling pathways that lead to the production of Type I interferons (IFNs) and other cytokines, which are crucial for antiviral defense [21, 22, 23, 24, 28, 29].

TLR3, TLR7, and TLR8 receptors recognize double‐stranded RNA (dsRNA) and ssRNA intermediates produced during viral replication, leading to the activation of downstream signaling cascades and the production of interferons [23, 30].

The interferon response is a key component of the innate immune defense against EV‐D68. Type I interferons (IFN‐α and IFN‐β) are produced in response to viral infection and induce the expression of ISGs that establish an antiviral state in host cells. Interferons bind to their receptors on the surface of infected and neighboring cells, triggering a signaling cascade that enhances the expression of ISGs, which inhibit viral replication and spread [23, 30].

**Figure 3:**
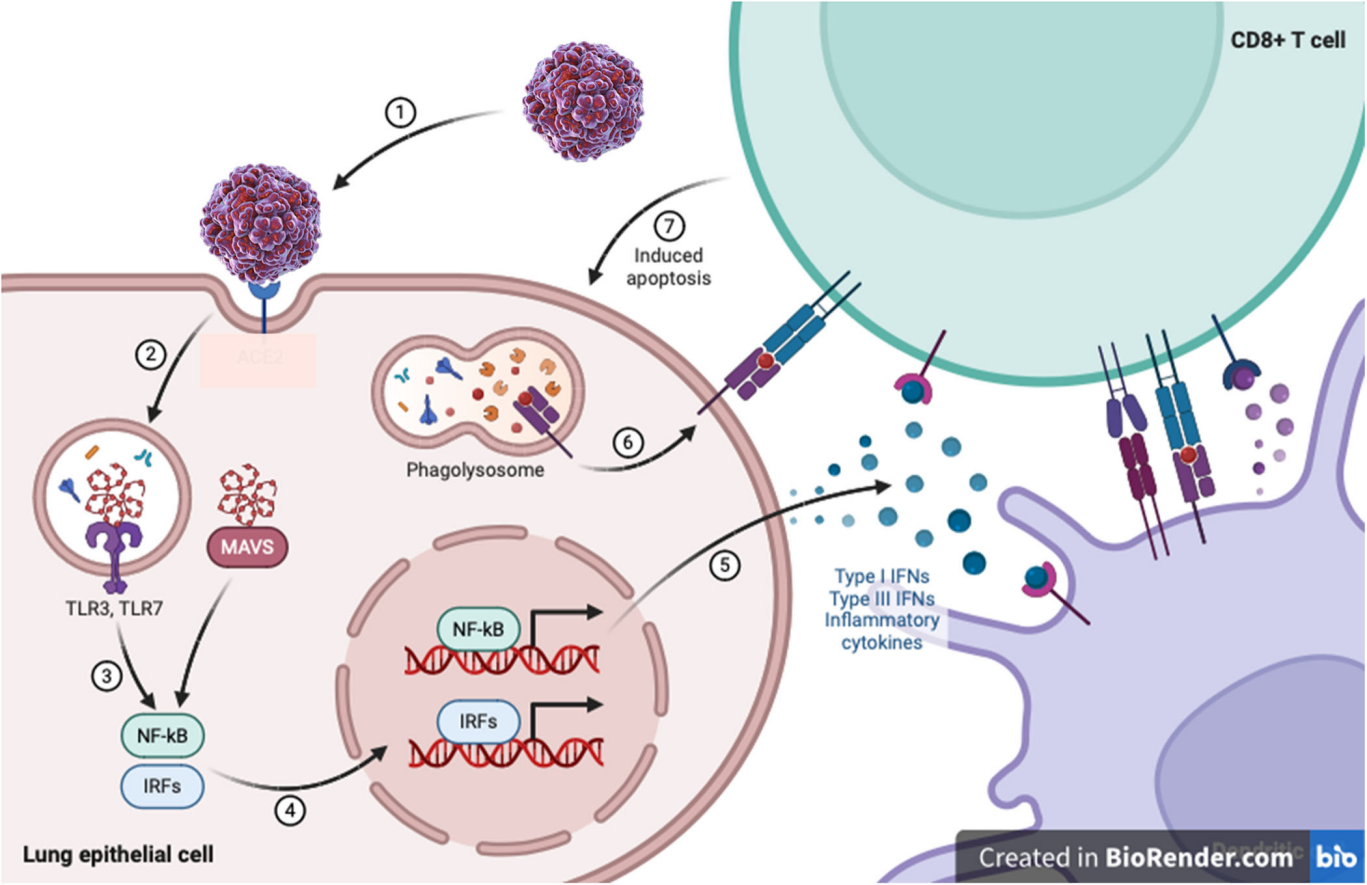
Innate immune modulation by RNA viruses.

A mouse model for EV‐D68 respiratory disease was developed to study the pathogenesis and immune response associated with the virus. The model involves infecting mice, typically neonatal or young adult mice, with EV‐D68 strains to mimic human respiratory infections. This model has been essential for understanding the viral lifecycle, host‐pathogen interactions, and the immune responses triggered by the infection. Infected mice exhibit symptoms similar to those observed in human infections, such as weight loss, lethargy, and respiratory distress. High levels of viral replication are observed in the lungs, leading to inflammation and damage to respiratory tissues. The model has helped delineate the innate and adaptive immune responses to EV‐D68, highlighting the roles of cytokines and immune cells in controlling the infection. This model has been used to test antiviral drugs and potential vaccines, providing a platform for preclinical evaluation of therapeutic strategies. Studies using mouse models of EV‐D68 respiratory disease have shown limited replication of viral RNA in the respiratory tract. However, the presence of viral RNA does not equate to the presence of an infectious virus, and to date, no animal model has demonstrated significant virus replication in the respiratory tract. This limitation underscores the need for developing more accurate models to study EV‐D68 pathogenesis and immune response [20, 31] (Figure 3).

Pathogen‐associated molecular patterns (PAMPs) in viral proteins and nucleic acids are recognized by cellular pathogen‐recognition receptors that include retinoic‐acid‐inducible gene I (RIG‐I), melanoma differentiation‐associated gene 5 and certain TLRs. PRR–PAMP interactions trigger signaling cascades that result in the activation of transcription factors, including interferon (IFN)‐regulatory factor 3 (IRF3) and nuclear factor‐κB (NF‐κB), which induce the production of Type I IFNs, IFN‐stimulated genes (ISGs) and pro‐inflammatory cytokines and chemokines. The specific process differs between antigen‐presenting cells, in which both the TLR pathway and the RIG‐I or MDA5 pathway are operative, and other cell types, in which only the RIG‐I or MDA5 pathway is present. Activation of PRR signaling induces an antiviral state in all cell types, and in antigen‐presenting cells, it can also induce the production of pro‐inflammatory cytokines and chemokines. This normally results in an innate antiviral response that controls infection until it is resolved by the adaptive immune response. MAVS, mitochondrial antiviral‐signaling protein; dendritic cells; dsRNA; IFN‐α receptor; IL, interleukin; NF‐κB, nuclear factor kappa‐light‐chain‐enhancer of activated B cells; ssRNA; TNF.

### 1.2 Adaptive Immunity

The adaptive immune response to EV‐D68 plays a critical role in the body's defense against the virus, particularly through the activation of both humoral and cellular immunity. When EV‐D68 infects a host, the immune system initiates a response where B cells are activated to produce antibodies targeting the virus's capsid proteins, such as VP1. These antibodies, particularly neutralizing antibodies, bind to the viral particles, preventing them from attaching to and entering host cells, which is crucial in limiting the spread of the virus and aiding in its clearance. The production of these antibodies is supported by CD4+ helper T cells, which release cytokines to enhance the overall immune response. Moreover, after the infection is cleared, memory B cells remain in the body, providing long‐term immunity by enabling a rapid and robust antibody response if the virus is encountered again [16, 30].

In addition to the humoral response, cellular immunity is vital in combating EV‐D68. CD8+ cytotoxic T cells are particularly important as they identify and destroy cells infected with the virus by recognizing viral peptides presented on MHC Class I molecules. This action helps to control viral replication and reduce the severity of the infection. Memory T cells are also formed during this process, allowing for quicker and more effective responses upon re‐exposure to EV‐D68. Despite these protective mechanisms, the virus has evolved various strategies to evade the adaptive immune system, such as antigenic variation, which complicates the development of effective vaccines and therapeutic strategies. Understanding these adaptive immune responses is crucial for advancing the design of vaccines and monoclonal antibody (mAb) therapies aimed at preventing and treating EV‐D68 infections [16, 30].

Serological study of patient samples provides evidence of adaptive immune responses triggered by EV‐D68. An analysis of the cerebrospinal fluid (CSF) of patients with AFM revealed a higher presence of antibodies specific to Picornaviridae compared to control patients. This was mostly due to the dominance of the Enterovirus genus. In addition, ELISA validated the presence of EV‐D68‐specific VP1 capsid protein antibodies in 85% of AFM patients. The presence of EV‐D68‐specific binding antibodies in the CSF of patients with AFM using ELISA has been described [32]. The frequency of EV‐D68 was examined in the United States by collecting serum samples from 2012 to 2013, which predates the first significant EV‐D68 epidemic. The findings of the study indicate that EV‐D68 infection may have been widespread in the United States before the outbreak in 2014 [33]. In a study conducted in Taiwan, it was shown that the production of neutralizing antibodies specific to EV‐D68 increased with age, indicating exposure to the virus over time. Specifically, 18% of toddlers aged 1–2 years tested positive for EV‐D68, whereas all 16–49‐year‐olds tested positive [34]. The specificity of these reactions to EV‐D68 remains uncertain because other investigations have shown substantial cross‐reactivity among related EVs. The study conducted by Rosenfeld et al. provided evidence that antibodies produced against recent isolates of EV‐D68 (2009, 2014, and 2018) in mice and guinea pigs exhibit significant cross‐reactivity with the poliovirus Type 1/Mahoney strain [35]. It is noteworthy that healthy human sera obtained from adults exhibited neutralization activity against EV‐D68, EV‐A71, and Poliovirus P1/Mahoney, indicating the possibility of cross‐neutralization across several enterovirus species. The neutralization of EV‐D68 by antibodies specifically generated against poliovirus and vice versa was validated in the mouse model [35]. Most neutralizing antibodies have been seen to specifically target EV‐D68 VP1 [36, 37, 38, 39]. However, it has been demonstrated that some antibodies could cross‐neutralize across several clades [39]. The isolation and characterization of many mAbs targeting EV‐D68 VP1 were conducted by Vogt et al. A total of 60 mAb was obtained from 12 individuals who had been previously infected with EV‐D68 during the 2014 epidemic. Among these subjects, 11 were infected with EV‐D68 from Clade B1, while 1 person was infected with EV‐D68 from Clade A1. The mAb were then evaluated for their ability to neutralize and bind to EV‐D68. Western blot analysis revealed that 7 out of the 60 mAbs exhibited binding to EV‐D68 VP1. Among these seven mAb (EV‐48, EV‐46, and EV‐40), cross‐clade neutralization was observed. mAbs EV‐48 and EV‐46 demonstrated cellular protection against infection caused by an isolate derived from Clade D (US/KY/14‐18953). In addition, EV‐40 exhibited cross‐clade neutralization by neutralizing the prototypic Fermon strain. Thirteen additional mAbs, which were not verified as anti‐VP1 antibodies, demonstrated the capability to inactivate the Clade D (four mAbs) and Fermon (nine mAbs) strains [39]. The neutralization of poliovirus infection in cell culture has been shown by anti‐EV‐D68 neutralizing antibodies, indicating the occurrence of heterotypic neutralization. However, it is probable that the effectiveness of heterotypic neutralization is decreased compared to type‐specific neutralizing antibodies [35].

Insufficient data exist on the characteristics and specificity of T cell responses triggered by EV‐D68 over the course of infection. In their study, Grifoni et al. used computational methods to illustrate that CD4+ T cell epitopes have a predominant localization inside VP1 and other structural proteins [40]. Rajput et al. found that mice infected with EV‐D68 generated greater amounts of IL‐17 compared to mice similarly infected with RV‐A. This suggests that the infection stimulates the development of Th17 and T cells that are unique to EV‐D68 [27]. The study conducted by Kreuter et al. revealed a significant presence of T lymphocytes in both the spinal cord and brain of a pediatric patient who succumbed to meningomyeloencephalitis induced by EV‐D68. Previous studies have shown that the presence of perforin, a biomarker associated with CD8 T cell function, was seen in the tissues, suggesting that infection is likely to elicit CD8 T cell responses specific to EV‐D68 [41]. Further research is required to comprehend the kinetics and characteristics of EV‐D68‐specific CD4 and CD8 T cells that are generated during infection, as well as the significance of these immune responses in providing protection.

### 1.3 Viral Evasion Mechanisms

Studies indicate that EV‐D68, like other EVs, interferes with the induction of Type I IFN responses. Multiple viral proteins, both structural and nonstructural, have been linked to dampening the innate immune response by reducing Type I IFN signaling. Kang et al. demonstrated that the capsid protein VP3 inhibits IRF7 phosphorylation, nuclear translocation, and ubiquitination by TRAF6 via competitive inhibition, resulting in interferon transcription suppression [42]. The nonstructural protein 3D polymerase is a key viral protein during viral replication; it has also been linked to mitochondrial dynamics and Type I interferon expression suppression. Yang et al. found that the 3D polymerase interacts with PGAM5 and upregulates the mitofusin 2 protein, altering the mitochondrial shape and inhibiting the RIG‐I receptor pathway, which leads to IFN‐β production [43]. EV‐D68 was demonstrated to upregulate suppressor of cytokine signaling 3 (SOCS3), which inhibits STAT3 phosphorylation and thereby suppresses the production of downstream ISGs [44].

Two prominent EV‐D68 proteases, the 2A and 3C proteases, have been linked to the evasion of innate immune responses. Kang et al. found that during EV‐D68 infection, 2A protease cleaves TRAF3, a critical protein in the development of Type I IFN, suppressing interferon production [42]. Others have shown that 2A protease suppresses stress granule formation, which contributes to IFN signaling [45]. Xiao et al. revealed that 3C protease cleaves both RIG‐I and TRIM25, ubiquitinating RIG‐I, which is required for receptor activation [46]. EV‐D68 3C protease has been shown to bind MDA5 and block its interaction with MAVS [47]. 3C protease inhibits IFN production by cleaving IRF7 and TIR‐domain‐containing adapter‐inducing interferon‐β (TRIF), inhibiting IRF3 activation, and blocking NF‐κB signaling [48, 49]. Collectively, these results indicate that innate IFN responses are activated early in infection, but the evasion of these innate responses allows EV‐D68 to establish a productive infection in certain individuals, leading to disease development.

EV‐D68 depends on its own protease, 3C, to directly cleave STAT1, a crucial component in the IFN signaling pathway. This disruption hinders the IFN‐mediated antiviral response, as observed in the study [50]. Prior research on human EVs has not observed any direct cleavage of the STAT1 protein as a means to evade cellular immune defenses. Nevertheless, some enteroviral 3C proteins are unable to cleave STAT1 [50].

EV‐D68 employs several mechanisms to evade the immune response:

#### 1.3.1 Cleavage of Host Proteins

EV‐D68 produces viral proteases such as 2A pro and 3C pro that cleave crucial host proteins, impairing the host's antiviral response. Specifically, 2A pro cleaves the eukaryotic initiation factor 4G (eIF4G), which is essential for the cap‐dependent translation of host mRNAs. This cleavage shuts down host protein synthesis while allowing viral RNA translation through an internal ribosome entry site (IRES)‐dependent mechanism [51, 52].

The mechanism by which EV‐D68 cleaves host proteins to evade the immune response involves the activity of viral proteases, primarily 2A pro and 3C pro.

##### 1.3.1.1 Cleavage of eIF4G

The eIF4G is a key component of the eIF4F complex, which is essential for the cap‐dependent translation of host mRNAs. EV‐D68 uses its protease 2A to cleave eIF4G. This cleavage disrupts the formation of the eIF4F complex, effectively shutting down the host cell's cap‐dependent translation machinery. As a result, the host's ability to produce antiviral proteins is severely impaired, allowing the virus to hijack the host's translational machinery for its own IRES‐dependent translation [51, 52, 53].

##### 1.3.1.2 Disruption of Host Protein Synthesis

By targeting eIF4G, EV‐D68 ensures that the host cell's ribosomes preferentially translate viral RNAs instead of host mRNAs. This selective translation not only promotes viral replication but also suppresses the host's immune response by reducing the production of proteins involved in antiviral defense [51].

##### 1.3.1.3 Cleavage of Other Host Proteins

In addition to eIF4G, EV‐D68's proteases also cleave other host proteins involved in immune signaling and cellular stress responses. For instance, the viral protease 3C pro cleaves components of the innate immune signaling pathways, such as MAVS, TRIF, and IRF family members. This cleavage disrupts the host's ability to mount an effective antiviral response by interfering with the production and signaling of Type I interferons and other cytokines [51].

##### 1.3.1.4 Impact on Cellular Structures

EV‐D68 infection can lead to the formation and subsequent disassembly of stress granules and processing bodies, which are involved in mRNA storage and degradation. The dynamic manipulation of these structures helps the virus control the stability and translation of both host and viral RNAs during different stages of infection.

By cleaving eIF4G, EV‐D68 inhibits host cell protein synthesis, redirecting the translational machinery to synthesize viral proteins. This not only facilitates viral replication but also reduces the production of antiviral proteins that depend on cap‐dependent translation.

Picornaviruses interfere with RNA decay pathways, protecting their RNA from degradation. For example, the virus can inhibit the activation of RNase L, an enzyme that degrades viral and cellular RNA upon activation by dsRNA intermediates. This inhibition helps the virus evade innate immune defenses [51].

EVs, including EV‐D68, disrupt the nucleocytoplasmic trafficking of host cell splicing factors, affecting RNA splicing and mRNA metabolism. This can lead to the production of defective mRNAs and proteins, further hampering the host's immune response [54].

EV‐D68 can modulate the host's innate immune signaling pathways. The virus encodes proteins that interfere with the host's antiviral signaling, including pathways mediated by interferons and other cytokines. By inhibiting these pathways, EV‐D68 can reduce the host's ability to mount an effective antiviral response. EV‐D68 infection increases the expression of SOCS3, which inhibits the JAK‐STAT3 pathway. This inhibition reduces the phosphorylation of STAT3, ultimately suppressing the production of ISGs that are crucial for antiviral responses [44].

The viral protein VP3 inhibits the TRAF6‐induced ubiquitination of IRF7. By preventing IRF7 activation, EV‐D68 reduces the production of Type I interferons, weakening the host's antiviral response [55].

The viral protease 3C pro cleaves signal transducer and activator of transcription 1 (STAT1), a key factor in Type I interferon signaling. This cleavage disrupts the interferon response, allowing the virus to evade immune detection [50].

EVs can also modulate autophagy, a cellular process involved in the degradation of cytoplasmic components. Autophagy is connected to RNA decay pathways and is activated during enterovirus infection. By manipulating autophagy, EV‐D68 may enhance its replication and persistence within host cells [56, 57, 58].

### 1.4 Vaccine and Therapeutic Developments

EV‐D68 vaccines, mAbs, and antiviral treatments are not yet authorized. The existing recommendations for the treatment of EV‐D68 include providing supportive care with asthma control, as well as addressing bronchoreactivity using bronchodilators and steroids, if deemed required. The use of commercial pooled immunoglobulins (Igs) has been seen in instances with EV‐D68‐associated AFM due to its notable presence of anti‐EV‐D68 antibodies, which have the potential to provide passive immunity. In addition, pooled Igs exhibit immunomodulatory features. Administering Igs promptly following EV‐D68 infection in a newborn mouse model has shown its efficacy in preventing paralysis [47]. Vogt et al. produced two mAb candidates that have shown a high level of effectiveness in neutralizing EV‐D68 [39]. Numerous promising antiviral candidates have been found via extensive screening of chemicals for their efficacy against EV‐D68 [59]. The selective serotonin reuptake inhibitor fluoxetine, which has been licensed by the FDA, has been seen to have antiviral action against circulating strains of EV‐D68 in vitro. However, retrospective research conducted in 2018 on non‐randomized usage in human instances of AFM did not provide any evidence of its effectiveness [60, 61]. The potential of the tiny molecule guanidine has been shown in mice models; nevertheless, the underlying mechanism remains unidentified at now. Telaprevir, a protease inhibitor licensed by the FDA, has shown the ability to limit the replication of EV‐D68 by binding irreversibly to the EV‐D68 2A protease. Telaprevir demonstrated enhanced paralysis results in a murine model. Although pocapavir and pleconaril, which are antiviral drugs specifically developed for EVs, did not show any effectiveness against EV‐D68, other possibilities, such as rupintrivir and V7404, which are protease inhibitors with EC50s as low as 0.0015–0.0051 μM, require additional investigation as potential antiviral candidates with in vitro EV‐D68 activity [62]. Finally, researchers are now working on developing vaccine candidates for EV‐D68 as a preventative measure against future outbreaks of EV‐D68 respiratory illness and AFM, if the number of patients continues to increase. Zhang et al. used an EV‐D68 VLP vaccine including *P. pastoris*, which co‐expressed the precursor P1 protein and 3CD protease. This vaccine effectively shielded newborn mice against EV‐D68 infection, both from vaccinated dams and from mice that received anti‐VLP sera passively [63, 64]. The bivalent EV‐D68/EV‐A71 mucosal vaccine developed by Lin et al. demonstrated the ability to elicit significant levels of neutralizing antibodies, specifically IgG and IgA, against EV‐D68 and EV‐A71. These antibodies effectively neutralized multiple subtypes of EV‐D68 and EV‐A71, thereby providing protection to neonatal mice against fatal infections caused by EV‐A71 and EV‐D68 [65, 66]. The study conducted by Krug et al. [65] provided evidence that a potential vaccination targeting EV‐D68 VLP induced significant production of neutralizing antibodies against several EV‐D68 clades in both murine and nonhuman primate models. An investigation into the optimal viral inactivation reagents, vaccine adjuvants, and route of vaccination in mice to optimize an inactivated whole‐virion vaccine against EV‐D68 was recently reported [66]. Further trials and investigations are necessary to comprehensively understand the efficacy of antivirals and vaccines in preventing EV‐D68 infection and mitigating its severe symptoms.

### 1.5 Future Perspectives

The ongoing challenge of accurately modeling human EV‐D68 infection in animal systems has been a significant barrier in the study of this virus. Current models lack immune competence, failing to replicate the full spectrum of human disease progression. Looking forward, there is a critical need to develop genetically modified mouse models or other alternative animal models that can better support virus replication and pathogenesis studies, thereby providing a more reliable platform for research.

Another major gap in our understanding of EV‐D68 is the specific cellular receptors the virus uses to gain entry into host cells. Despite extensive research, these receptors remain poorly characterized. Future efforts should focus on employing advanced techniques such as CRISPR‐Cas9 screening and proteomics to identify and validate these receptors, which could lead to significant breakthroughs in understanding the virus's entry mechanisms.

Our knowledge of how EV‐D68 interacts with and evades the host immune system is also incomplete. Although we understand the basics of the innate and adaptive immune responses to EV‐D68, including the roles of T cells and the potential for cross‐reactivity with other EVs, much remains to be explored. Detailed studies are needed to uncover the full extent of these interactions and the virus's evasion strategies.

Vaccine development is another area where progress has been stymied, largely due to the inadequacy of current animal models for testing efficacy. Future strategies should include the development and testing of multivalent vaccines targeting multiple enterovirus serotypes. In addition, exploring novel vaccine platforms, such as mRNA‐based vaccines, could offer new avenues for protection against EV‐D68.

In the realm of antiviral therapeutics, the data are similarly limited. To address this, there is a pressing need to screen existing antiviral compounds for activity against EV‐D68 and to develop new therapies that target the virus's unique biological mechanisms.

Finally, our understanding of EV‐D68's epidemiology, particularly its association with diseases like AFM, is hindered by a lack of long‐term data. Comprehensive longitudinal epidemiological studies are essential to track infection patterns, mutation rates, and the emergence of new strains over time.

Addressing these gaps will require a multidisciplinary approach, integrating insights from virology, immunology, molecular biology, and clinical research. Collaborative efforts across these fields will be crucial in advancing our understanding of EV‐D68 and developing effective strategies to combat this re‐emerging pathogen.

## Author Contributions

Asif Naeem contributed to study design, data analysis, results discussion, and manuscript writing and review. Muhammad Bashir Bello contributed to manuscript writing. Mohammad Bosaeed contributed to manuscript writing and review. All authors read and approved the final manuscript.

## Conflicts of Interest

The authors declare no conflicts of interest.

## Data Availability

The authors have nothing to report.
